# Function of Protein Kinases in Leaf Senescence of Plants

**DOI:** 10.3389/fpls.2022.864215

**Published:** 2022-04-25

**Authors:** Fengbo Yang, Yuchen Miao, Yuyue Liu, Jose R. Botella, Weiqiang Li, Kun Li, Chun-Peng Song

**Affiliations:** ^1^State Key Laboratory of Crop Stress Adaptation and Improvement, School of Life Sciences, Henan University, Kaifeng, China; ^2^State Key Laboratory of Cotton Biology, Henan Joint International Laboratory for Crop Multi-Omics Research, School of Life Sciences, Henan University, Kaifeng, China; ^3^School of Agriculture and Food Sciences, University of Queensland, Brisbane, QLD, Australia

**Keywords:** leaf senescence, protein kinase, phytohormone, reactive oxygen species, calcium signal, metabolism

## Abstract

Leaf senescence is an evolutionarily acquired process and it is critical for plant fitness. During senescence, macromolecules and nutrients are disassembled and relocated to actively growing organs. Plant leaf senescence process can be triggered by developmental cues and environmental factors, proper regulation of this process is essential to improve crop yield. Protein kinases are enzymes that modify their substrates activities by changing the conformation, stability, and localization of those proteins, to play a crucial role in the leaf senescence process. Impressive progress has been made in understanding the role of different protein kinases in leaf senescence recently. This review focuses on the recent progresses in plant leaf senescence-related kinases. We summarize the current understanding of the function of kinases on senescence signal perception and transduction, to help us better understand how the orderly senescence degeneration process is regulated by kinases, and how the kinase functions in the intricate integration of environmental signals and leaf age information.

## Introduction

Leaves are the main organs for photosynthesis in plants and places for water transpiration and carbon dioxide (CO_2_) exchange (Efroni et al., 2010; Kalve et al., 2014; Thomas and Ougham, 2014). Leaf senescence involves the orderly breakdown of cellular structures (including chloroplasts), and hydrolysis of macromolecules (proteins, carbohydrates, lipids, and nucleic acids, etc.) to generate nutrients, which will be reallocated into developing fruits, seeds or storage tissues which depending on the life cycle of species (Rivero et al., 2007; Watanabe et al., 2013, 2018; Kim J. et al., 2016; Thomas and Ougham, 2016). Leaf senescence occurs at various levels including the deterioration and death of cells, tissues, or the whole leaf, and it is a critical process for plant survival as well as ensuring the successful reproduction of the next generation. Meanwhile, leaf senescence is a highly complex process involving orderly and highly coordinated degeneration and remobilization, which are tightly regulated by many genes, including NAC, WRKY and other transcription factors (Koyama, 2018; Woo et al., 2018; Ahmad and Guo, 2019; Jan et al., 2019).

Leaf senescence is generally controlled by plant age and developmental stage, additionally internal factors and external environmental signals also trigger the onset of senescence. The internal factors mainly include endogenous hormones levels, source-sink relationships, and carbon-nitrogen resource allocation. The external environmental signals mainly refer to abiotic and biotic stresses, such as high salinity, low water status, soil nutrient deficiency, unfavorable light regimes, extreme temperature changes, and pathogen infection, all of them are involved in triggering the aging process of leaf. Since there are many different signals perception and transduction pathways actions coordinately control leaf senescence, it is reasonable to assume that there are significant overlaps and cross-talks between different signaling pathways in the regulation of leaf senescence, therefore, leaf senescence is regulated by a complex and precise network (Buchanan-Wollaston et al., 2003; Lim et al., 2007; Jibran et al., 2013; Woo et al., 2019).

In this review, we will summarize in-depth synopsis of protein kinases in the regulation of leaf senescence. We will present the function of protein kinases in different signal transduction pathways and regulatory networks involved in leaf senescence, to explore the molecular mechanisms of how internal and external senescence cues are perceived and ultimately lead to transcriptional level regulation of senescence associated genes and thus the execution of leaf senescence.

## Age-Dependent and Stress-Induced Leaf Senescence in Plants

Leaf senescence is generally defined as age-dependent and the stress-induced senescence. Plants display senescence syndrome even under optimal growth conditions, which is the best known as age-dependent senescence (Buchanan-Wollaston et al., 2005). The initiation of age-dependent leaf senescence is tightly related to the developmental cues of plants, and leaves start senescence process when they reach to maturation stage, and the change of source-sink relationship may be one of the factors to trigger age-dependent leaf senescence (Thomas, 2013; Rankenberg et al., 2021). In addition, leaf senescence can also be induced by various signals, including hormonal, nutritional status, abiotic and biotic stresses during development process (Schippers, 2015; Schippers et al., 2015). Stress-induced leaf senescence only occurs in mature plants, and Arabidopsis juvenile leaves do not show senescence symptoms under ethylene treatment, and the length of vegetative growth stage is shortened under stress conditions (Jing et al., 2005; Miryeganeh, 2021).

Previous studies have found that although the initiation signals of leaf senescence are different, plant leaves show similar morphological, physiological, biochemical and transcriptional changes, which are the consequence of the similar signal transduction systems both in age-dependent and stress-induced leaf senescence (Guo et al., 2004; Guo and Gan, 2005). Meanwhile, many senescence associated genes (*SAGs*) are involved in both age-dependent senescence and stress-induced senescence (van der Graaff et al., 2006; Guo and Gan, 2012), therefore, the underlying regulatory mechanisms may overlap (Kim H. J. et al., 2016). Protein kinases, such as RPK1 and SnRK1, are also involved in both age-dependent senescence and stress-induced senescence (Lee et al., 2011; Cho et al., 2012; Kim et al., 2017; Koo et al., 2017). We mainly focus on function of protein kinases in stress-induced leaf senescence in this review.

## Receptor-Like Kinases in Leaf Senescence

Plant typical receptor-like kinases (RLKs) are transmembrane proteins typically containing an N-terminal extracellular domain for ligand binding and a C-terminal intracellular kinase domain to phosphorylate downstream components. RLKs usually form homo- or hetero-dimers (Jose et al., 2020). Ligand binding to the extracellular domain induces trans-phosphorylation of the monomers before transmitting the signal to downstream components by phosphorylation to activate the regulatory network. The Arabidopsis (*Arabidopsis thaliana*) genome contains more than 600 RLKs, including approximately 150 receptor-like cytoplasmic kinases (RLCKs) that lack the extracellular domain and associate with receptor complexes to function (Liang and Zhou, 2018). RLKs have important function in diverse biological processes, including plant growth and development, self-incompatibility, hormone perception and resistance to biotic and abiotic stresses (Berger, 2009; Chae et al., 2009; Vaid et al., 2013; Antolín-Llovera et al., 2014; Ye et al., 2017). Extracellular RLK domains are classified into more than 20 classes, including S-domains, leucine-rich repeats (LRR), epidermal growth factor-like (EGF), lectin-like, tumor-necrosis factor (TNF), and pathogenesis related-5 protein (PR5), etc. Although a large number of RLKs have been identified in plants, initial studies mainly focused on expression patterns and biochemical analyses, therefore, functional studies of their signal transduction pathways are still inadequate, and most RLKs are still “orphan,” i.e., the ligands and downstream targets of most RLKs in Arabidopsis and Rice (Shiu et al., 2004), and a group of RLKs as cell surface receptors for root meristem growth factors are still unknown (Yu and Luan, 2016), which need further investigation.

Cell surface-localized RLKs can sense and transmit a variety of signals in response to environmental stresses and play essential roles in a wide range of physiological and developmental processes, including leaf senescence. The roles of a number of key RLKs in regulating leaf senescence have been characterized in various plant species (Table 1). A LRR-RLK from bean (*Phaseolus vulgaris*) was named senescence-associated receptor-like kinase (SARK) because its mRNA and protein levels increased during leaf senescence (Hajouj et al., 2000). PpSARK (*Physcomitrella patens* senescence-associated receptor-like kinase), with high homology to the bean SARK, is involved in the regulation of moss (*Physcomitrella patens*) senescence (Li et al., 2018). Another LRR-RLK gene involved in the regulation of leaf senescence was isolated in the soybean (*Glycine max*) and named *Glycine max* senescence-associated receptor-like kinase (GmSARK). Downregulation of *GmSARK* in transgenic soybean resulted in delayed leaf senescence while overexpressing lines showed increased senescence rates (Li X. et al., 2006). AtSARK (*A. thaliana* senescence-associated receptor-like kinase), the homolog of GmSARK, positively regulates leaf senescence in Arabidopsis, with overexpression of *AtSARK* leading to premature of leaf senescence, whereas down-regulation caused delayed leaf senescence (Xu et al., 2011). In Arabidopsis, RPK1 (receptor protein kinase 1) has a positive role in age-dependent and ABA (abscisic acid)-induced leaf senescence (Lee et al., 2011), mediated by the NADPH oxidase RbohF (respiratory burst oxidase homolog protein F) (Koo et al., 2017). Another Arabidopsis LRR-RLK, SERK4 (somatic embryogenesis receptor kinase 4) which is induced during leaf senescence as well as by several abiotic stresses has a negative role in the regulation of leaf senescence (Li et al., 2019; Yu et al., 2019; Zhou et al., 2019). The rice (*Oryza sativa*) senescence-induced receptor-like kinase (OsSRLK) is involved in phytohormone-mediated chlorophyll degradation under dark-induced senescence (Shin et al., 2019). Phosphorylation levels of a leucine-rich repeat malectin kinase 1 (LMK1) were strongly affected by high C/low N-nutrient stress and overexpression of LMK1 induced cell death in *Nicotiana benthamiana* leaves (Li X. et al., 2020).

**TABLE 1:** Receptor-like kinases (RLKs) function in the regulation of leaf senescence.

Kinase name	Species	Performance during leaf senescence	Function	Role	References
PvSARK	*P. vulgaris*	mRNA and protein levels increased under natural- and induced-leaf senescence	Unknown	Unknown	Hajouj et al., 2000
PpSARK	*P. patens*	The gain-function- mutants display insensitive to ABA induced leaf senescence	Regulates high salt and ABA responses	Negative	Li et al., 2018
GmSARK	*G. max*	*GmSARK* knock-down plants show delay leaf senescence and the over-expression lines display early leaf senescence	Regulating chloroplast development and chlorophyll accumulation	Positive	Li et al., 2006
AtSARK	*A. thaliana*	*AtSARK*-overexpressing seedlings display precocious leaf senescence	Regulating leaf senescence through synergistic actions of auxin and ethylene	Positive	Xu et al., 2011
RPK1	*A. thaliana*	*rpk1* mutants exhibit delayed age-dependent and ABA-induced senescence symptoms	Regulates the expression of *SAGs* and ABA-inducible genes	Positive	Lee et al., 2011; Koo et al., 2017
SERK4	*A. thaliana*	*SERK4* was up-regulated during leaf senescence, and *serk4* mutants display a significant early leaf senescence	Regulates ROS generation, Ca^2+^ homeostasis and cell death	Negative	Li et al., 2019; Yu et al., 2019; Zhou et al., 2019
OsSRLK	*O. sativa*	*OsSRLK* is upregulated in senescing rice leaves. The detached leaves of *srlk* contained more green pigment during dark-induced senescence	Participates in phytohormone-mediated chlorophyll degradation under dark-induced senescence	Positive	Shin et al., 2019
LMK1	*N. benthamiana*	Response to high C/low N-nutrient stress and overexpression of *LMK1* induces cell death in *N. benthamiana* leaves	Unknown	Positive	Li et al., 2020
AtWAKL10	*A. thaliana*	*wakl10* mutants display earlier leaf senescence and the overexpression plants delay the aging process	Unknown	Negative	Li et al., 2021
HvLysMR1	*H. vulgare*	Transcript accumulates during leaf senescence	Unknown	Unknown	Ouelhadj et al., 2007
OsSIK2	*O. sativa*	*OsSIK2*-overexpression seedlings exhibit early leaf development and delayed dark-induced senescence, while *sik2* mutants show opposite phenotype	Enhances plants tolerance to abiotic stress	Negative	Chen et al., 2013
CRK5	*A. thaliana*	*crk5* mutants show accelerated leaf senescence	Regulates the accumulation of ROS, ethylene, SA	Negative	Burdiak et al., 2015
OsBBS1	*O. sativa*	*bbs1* seedlings are hypersensitive to salt and show premature leaf senescence	ROS accumulation and cell death	Negative	Zeng et al., 2018

Besides LRR-RLKs, other RLKs also have roles in the regulation of leaf senescence. The WAK-like kinases (WAKLs) belong to EGF-RLKs, and one of WAKLs, AtWAKL10 is induced by ABA, JA, and SA, and it negatively modulates the leaf senescence progression, the *atwakl10* mutants display accelerated leaf senescence and *AtWAKL10* overexpression plants show opposite phenotype (Li et al., 2021). The expression of *HvLysMR1*, a barley (*Hordeum vulgare*) lysine motif RLK is induced by heavy metal and calcium ionophore A23187 treatment as well as leaf senescence (Ouelhadj et al., 2007). OsSIK2 (*O. sativa* stress-induced protein kinase gene 2), an S-domain receptor-like kinase in rice (*O. sativa*), is expressed mainly in leaf and sheath, and induced by several abiotic stresses. Transgenic rice plants over-expressing *OsSIK2* exhibited delayed dark-induced leaf senescence (Chen et al., 2013). Mutation of the Arabidopsis cysteine-rich receptor-like kinase CRK5, produce accelerated senescence correlated with accumulation of reactive oxygen species (ROS), ethylene and salicylic acid (Burdiak et al., 2015). OsBBS1/OsRLCK109 encodes a RLCK in rice, it is involved in salt stress response and leaf senescence, seedlings of *bbs1* (*bilateral blade senescence 1*) mutants are hypersensitive to salt and show premature leaf senescence phenotype (Zeng et al., 2018).

## Hormone Signaling and Intracellular Second Messengers Regulated Protein Kinases Involved in Leaf Senescence

**FIGURE 1:**
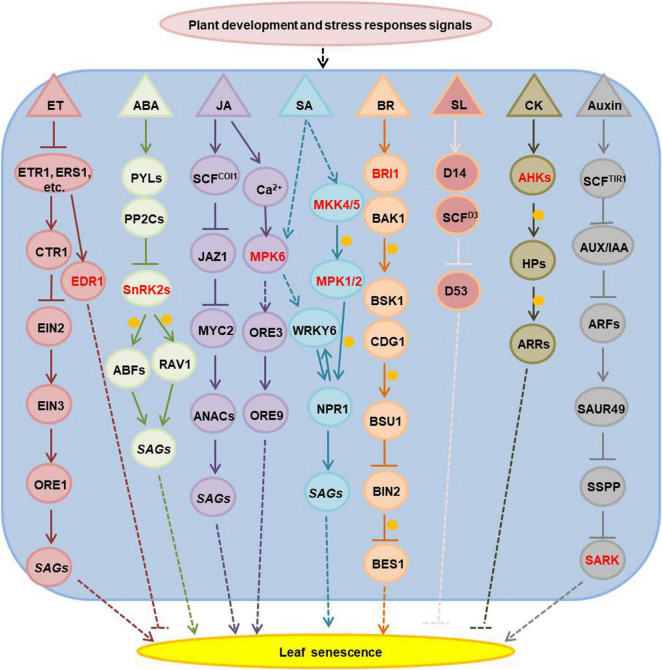
The senescence-associated kinases functioned in hormones-regulated leaf senescence. The kinases are marked with red; arrows show that the process is promoted, and T-bars mean the process is inhibited; the solid lines indicate direct reported relationship, and the dotted lines indicate indirect reported relationship; the orange circles indicate phosphorylation. ET, ethylene; ABA, abscisic acid; JA, jasmonic acid; SA, salicylic acid; BR, brassinosteroids; SL, strigolactone; CK, cytokinins; ETR1, ethylene response 1, it is one of the five ET receptors; ERS1, ethylene response sensor 1, one of ET receptors; CTR1, constitutive triple response 1, homologous to the RAF family of serine/threonine protein kinases, a negative regulator in the ethylene signal transduction pathway, it interacts with the putative ethylene receptors ETR1 and ERS1; EIN2, ethylene insensitive 2, it acts downstream of CTR1 to regulate EIN3 positively; EIN3, ethylene-insensitive 3, a nuclear transcription factor that initiates downstream transcriptional cascades for ethylene responses; ORE1, ORESARA 1, it is a NAC-domain transcription factor and regulates senescence in leaves positively; *SAGs*, senescence associated genes; EDR1, enhanced disease resistance 1, a RAF family of serine/threonine protein kinases like CTR1, it has a negative role in ET signal pathway; PYLs, pyrabactin resistance 1-like family proteins, function as the ABA receptors; PP2Cs, the type 2C protein phosphatases, function as ABA co-receptors; SnRK2s, sucrose non-fermenting 1-related protein kinase 2 family proteins, they are activated by ABA and inhibited by PP2Cs; ABFs, ABA responsive element binding factor proteins, as leucine zipper transcription factors that bind to the ABA-responsive element (ABRE) motifs in the promoter region of ABA-inducible genes; RAV1, related to ABI3/VP1 1, an AP2/B3 domain transcription factor which is upregulated in ABA-induced leaf senescence; COI1, coronatine insensitive 1, JA receptor, it associates with AtCUL1, AtRbx1, and the Skp1-like proteins to assemble SCF^COI1^ ubiquitin-ligase complexes; JAZ1, jasmonate-zim-domain protein 1, it is degraded by SCF^COI1^ ubiquitin-ligase complexes under JA stimulus; MYC2, MYC-related transcriptional activator 2; ANACs, NAC domain-contained transcription factors; MPK6, mitogen-activated protein kinase (MAPK) 6; ORE3, ORESARA 3 or named as EIN2; ORE9, ORESARA 9, as a member of the F-box leucine-rich repeat family proteins, it is a proposed regulator of leaf senescence; MKK4/5, MAPK kinase 4/5; MPK1/2 (MAPK1/2), mitogen-activated protein kinase 1/2; BRI1, BR insensitive 1, encodes a plasma membrane localized leucine-rich repeat receptor kinase, as BR receptor; BAK1, BRI1-associated receptor kinase, as the BR co-receptor with BRI1, it is a leucine-rich receptor serine/threonine protein kinase; BSK1, BR-signaling kinase 1; CDG1, constitutive differential growth 1, is a receptor-like cytoplasmic kinase, belongs to RLCKVII subfamily; BSU1, BRI1 suppressor 1, encodes a serine-threonine protein phosphatase; BIN2, brassinosteroid-insensitive 2, a member of the ATSK (shaggy-like kinase) family; BES1, BRI1-EMS-suppressor 1, a key transcription factor involved in BR signaling, coordinates plant growth and stress responses; D14, is a receptor in the SL signaling pathway; SCF^D3^, as a member of the F-box leucine-rich repeat family of proteins, they are involved in SCF-dependent protein ubiquitination; D53, interacts with D14 in an SL-dependent manner, and it is shown to be degraded through the 26S proteasome pathway in a manner that requires the function of the F-box protein D3; AHKs, Arabidopsis histidine kinases, CK receptors, including AHK2, AHK3, and AHK4; HPs, histidine-containing phosphotransfer proteins; ARRs, Arabidopsis response regulators, including type-A and type-B ARR; TIR1, transport inhibitor response 1, encodes an auxin receptor, it contains leucine-rich repeats and an F-box and forms SCF (Skp-Cullin-F-box) complexes with ASK1 and CUL1; AUX/IAA, repressors of auxin-responsive transcription; ARFs, auxin-response factors; SAUR, small auxin upregulated RNA; SSPP, senescence suppressed protein phosphatase; SARK, senescence-associated receptor-like kinase.

Hormones are essential for plant development and stress responses, thus they have a significant role in the regulation of age-dependent and stress-induced leaf senescence. Ethylene, ABA, jasmonic acid, salicylic acid, brassinosteroids and strigolactone promote, while cytokinins and gibberellins inhibit leaf senescence (Jan et al., 2019; Woo et al., 2019; Chen et al., 2020). Many kinases involved in plant hormone signaling and have been associated to the regulation of leaf senescence (Figure 1 and Supplementary Table 1). The Arabidopsis EDR1 (enhanced disease resistance 1), which is an mitogen-activated protein kinase kinase kinase (MAPKKK), plays a negative role in the ethylene signaling pathway and *edr1* mutants show enhanced leaf senescence under ethylene treatment (Tang and Innes, 2002; Tang et al., 2005). A wheat (*Triticum aestivum*) ethylene receptor homolog (W-er1), with a histidine kinase domain, is induced during jasmonate and ABA triggered leaf senescence (Ma and Wang, 2003). The Arabidopsis SnRK2s (sucrose non-fermenting 1 related protein kinase 2), which have a positive function in the ABA signaling pathway, can phosphorylate ABA-responsive element binding factors (ABFs) and RAV1 (related to abi3/vp1 1) transcript factors to activate the expression of senescence associated genes in ABA-induced leaf senescence (Gao et al., 2016; Zhao et al., 2016). MPK6 (mitogen-activated protein kinase 6) has a regulatory role in both jasmonic acid- and salicylic acid- mediated leaf senescence (Yue et al., 2012; Chai et al., 2014; Zhang et al., 2016). The MKK4/5-MPK1/2 (mitogen-activated protein kinase kinase 4/5- MPK1/2) cascade regulates SA-induced leaf senescence through phosphorylation of NPR1 (non-expresser of PR genes 1) (Zhang et al., 2020). Mutants in BRI1 (Brassinosteroid insensitive 1), a component of the brassinosteroids receptor complex, show dark-green leaves and delayed senescence (He et al., 2007). Cytokinins are perceived by the histidine kinase receptors: AHK2, AHK3, and AHK4 (Arabidopsis His-kinase 2/3/4). Mutations in all three genes lead to shorter leaf longevity and loss of the ability to retain chlorophyll under cytokinin treatment in dark-induced leaf senescence (Riefler et al., 2006). AHK3 is the major cytokinin receptor involved in the control of leaf longevity by phosphorylation of ARR2 (a response regulator 2), an important transcription factor involved in the cytokinin signaling transduction pathway (Kim et al., 2006). Exogenous application of indole-acetic acid (IAA) negatively regulates leaf senescence (Kim et al., 2011), while Several SAURs (small auxin up-regulated RNA) are positive regulators of leaf senescence (Kant et al., 2009; Hou et al., 2013; Bemer et al., 2017). Therefore the detailed functions of auxin in leaf senescence remain controversial. Recent research showed that SAURs functions in accelerating the leaf senescence process via the activation of SARK-mediated leaf senescence signaling by suppressing SSPP (senescence suppressed protein phosphatase) (Wen et al., 2020).

**FIGURE 2:**
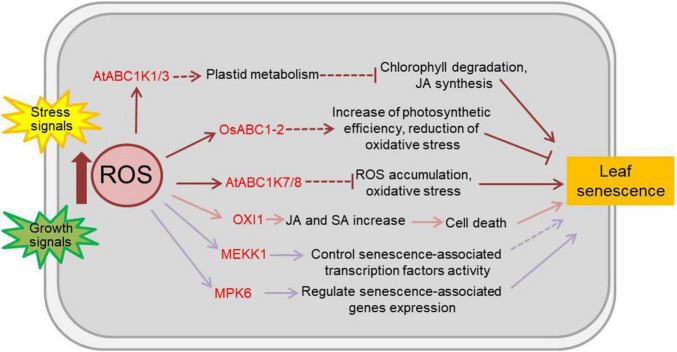
Effects of ROS on plant leaf senescence. Numerous abiotic and biotic stresses like salinity, heat, cold, nutrients, heavy metals, insects, pathogens, etc., resulting to the accumulation of ROS and the change of kinase activity. As the main and important second messenger in plants, ROS participate in a number of physiological responses and development process including leaf senescence. The kinases functioned in ROS-regulated leaf senescence are marked in red. Arrows show that the process is promoted, and T-bars mean the process is inhibited; solid lines indicate the direct reported relationship, and dotted lines indicate indirect reported relationship. OXI1, oxidative signal-inducible 1; ABC1 atypical kinase, activity of bc1 complex kinase; MEKK1, MAPK/ERK kinase kinase 1; MPK6, mitogen-activated protein kinase 6; JA, jasmonic acid; SA, salicylic acid.

Reactive oxygen species, comprised of singlet oxygen (^1^O_2_), superoxide radical (O_2_^–^), hydrogen peroxide (H_2_O_2_), and hydroxyl radical (HO), are naturally generated as metabolic by-products in chloroplasts, mitochondria, peroxisomes and the apoplast of plants. ROS are highly toxic due to their reactive properties, which result in severe damage to cellular macromolecules, such as lipids, proteins, and nucleic acids. However, ROS are also known to play important roles in sensing and signal transduction in response to various biotic and abiotic stimuli and during developmental processes in plants (Møller et al., 2007; Swanson and Gilroy, 2010; Waszczak et al., 2018). OXI1 (oxidative signal-inducible 1), a serine/threonine protein kinase of the AGC (cAMP-dependent, cGMP-dependent and protein kinase C) kinase family, is a downstream component of ROS signals and is activated by oxidative stress and wounding (Rentel et al., 2004). ^1^O_2_ can lead to programmed cell death through the action of OXI1 at high light levels. Arabidopsis OXI1 over-expressing lines display hypersensitivity to high light and early senescence even in normal light conditions (Shumbe et al., 2016; Beaugelin et al., 2019). Most plant ABC1 atypical kinase (ABC1K, activity of bc1 complex kinase) proteins are located in either chloroplasts or mitochondria and are involved in the response to stresses (Lundquist et al., 2012). OsABC1-2, a rice ABC1K protein, encodes a chloroplast envelope-localized protein primarily present in green tissues. The null *osabc1-2* mutants have small plant size and pale-green leaves, and the *OsABC1-2* overexpressing lines show enhanced tolerance to prolonged dark-induced leaf senescence (Gao et al., 2012). The Arabidopsis ABC1K7 and ABC1K8 are involved in ROS homeostasis (Manara et al., 2015). *ABC1K7* and *ABC1K8* are upregulated by ABA, and the single *abc1k7* and *abk1k8* mutants and the double *abc1k7 abk1k8* mutants exhibit faster senescence rate than wild type plant under ABA treatment (Manara et al., 2016). However, the Arabidopsis plastoglobules-localized kinases ABC1K1 and ABC1K3 play roles in the regulation of high light stress induced leaf senescence, with a ROS-independent manner. The *abc1k1* and *abc1k3* mutants display rapid chlorosis in high light stress, and the double mutants show slow and irreversible senescence-like phenotype in moderate light caused by increased levels of jasmonate biosynthesis and pheophytinase activity, which accelerate chlorophyll degradation (Lundquist et al., 2013). In addition, the MAPK (mitogen-activated protein kinase) cascade is the classical signal transduction pathway in response to ROS (Jalmi and Sinha, 2015), in which MEKK1 and MPK6 are activated by ROS and reported to be involved in the aging process of plant (Nakagami et al., 2006; Miao et al., 2007; Zhou et al., 2009). Kinases related to ROS-regulated leaf senescence are summarized in Figure 2 and Supplementary Table 2.

**FIGURE 3:**
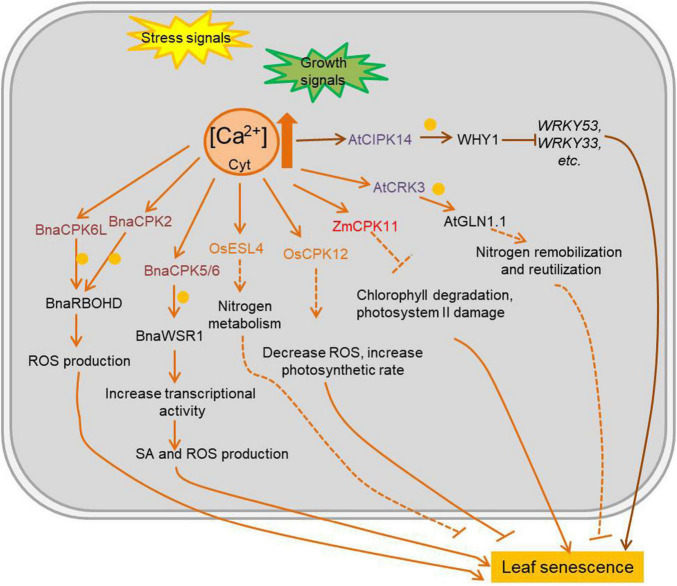
Ca^2+^ mediated plant leaf senescence in different conditions. Intracellular Ca^2+^ change rapidly during plant development and stress response, and it always acts as second messenger to activate Ca^2+^ sensing proteins. The kinases which are regulated by levels of Ca^2+^ and function in leaf senescence are marked in colors. Arrows show that the process is promoted, and T-bars mean the process is inhibited; blue circles indicate phosphorylation; solid lines indicate the direct reported relationship, and dotted lines indicate indirect reported relationship. CPKs/CDPKs, calcium-dependent protein kinases; CIPKs, CBL-interacting protein kinases; CBL, calcineurin B-like proteins; OsESL4, *O. sativa* early senescent leaf 4; AtCRK3, *Arabidopsis thaliana* CDPK-related kinase 3; BnaWSR1, *B. napus* WRKY regulating SA and ROS 1; AtGSR1, *Arabidopsis thaliana* glutamine synthase clone R 1; WHY1, WHIRLY1; JA, jasmonic acid; SA, salicylic acid.

Ca^2+^ is a ubiquitous second messenger with an important signaling role in various stresses and developmental processes. Except for rapid and/or spatially restricted expanding cell, the concentration of resting cytosolic Ca^2+^ ([Ca^2+^]) is kept approximately 100–200 nM, due to its potential toxicity at higher levels, but organelles and the extracellular space can reach millimolar Ca^2+^ concentrations. Consequently, a steep [Ca^2+^] concentration gradient is established between the cytosol and the different Ca^2+^ stores. One of the most intriguing aspects of Ca^2+^ signaling is the complex spatio-temporal patterns of Ca^2+^ influx, including concentration, amplitude, duration and oscillation induced in cells by various stimuli. Free Ca^2+^ is sensed and decoded by several types of Ca^2+^-binding proteins with EF-hand motifs. Calmodulin (CaM), a highly conserved eukaryotic protein with four EF-hand domains, is involved in the regulation of multiple interacting proteins (e.g., transcription factors). Calcineurin B-like (CBL) proteins are regulatory proteins without enzymatic activity *per se*, but they interact with specific CBL-interacting protein kinases (CIPKs), which are activated upon CBL binding (Steinhorst and Kudla, 2013). Calcium-dependent protein kinases (CDPKs or CPKs) have an N-terminal variable domain, a protein kinase domain, an auto-inhibitory junction domain, and a C-terminal calmodulin-like domain (Atif et al., 2019). Many calcium-related kinases are involved in the regulation of leaf senescence (Figure 3 and Supplementary Table 3). AtCIPK14 has an indirect negative effect in leaf senescence by phosphorylating the transcription factor WHY1 (WHIRLY1). Once phosphorylated by AtCIPK14, the accumulation of WHY1 increased in nucleus, promoting its binding to the promoter of *WRKY53* and thus decreasing the expression of several *SAGs* (Ren et al., 2017). ESL4 (early senescent leaf 4), a rice CDPK, is involved in nitrogen metabolism and leaf senescence, with *esl4* mutants showing premature leaf senescence when grown under low-nitrogen conditions (Xing et al., 2018). The rice OsCPK12 plays a role in leaf senescence by regulating ROS levels and photosynthetic rate (Wang B. et al., 2019). Overexpression of the maize *ZmCPK11* in Arabidopsis, improves salt tolerance by preventing salt-induced chlorophyll degradation and damage to photosystem II (Borkiewicz et al., 2020). The *Brassica napus* transcription factor BnaWSR1 binds to the promoter of *ICS1* (*isochorismate synthase 1*), *RbohD* (*respiratory burst oxidase homolog protein D*), and *SAG14* (*senescence associated gene 14*) to regulate their expression, resulting in the accumulation of SA and ROS during the leaf senescence process. BnaCPK5/6 (*B. napus* Calcium-dependent protein kinase 5/6) interacts with and phosphorylates BnaWSR1 (*B. napus* WRKY regulating SA and ROS 1) to enhance its transcriptional activity, thus BnaCPK5/6 is involved in cell death and leaf senescence (Cui et al., 2020). BnaCPK2 and BnaCPK6L (*B. napus* Calcium-dependent protein kinase 2; *B. napus* Calcium-dependent protein kinase 6) interact with and phosphorylate BnaRBOHD (*B. napus* Respiratory burst oxidase homolog D) both to enhance BnaRBOHD activity and generate more ROS in cell which would accelerate cell death and leaf senescence (Wang et al., 2018; Pan et al., 2019). A CDPK-related kinase (CRK) AtCRK3, is involved in regulation of leaf senescence in Arabidopsis by phosphorylating the cytosolic glutamine synthetase AtGLN1;1/AtGSR1 (*A. thaliana* glutamine synthase clone R 1), important for nitrogen remobilization and reutilization during leaf senescence (Li et al., 2006).

Reactive oxygen species, Ca^2+^ and phytohormone are involved in the regulation of almost all growth stages and stress responses in plants. The phytohormones function always integrated with ROS, Ca^2+^, and it has been studied extensively (Shabala et al., 2016; Choi et al., 2017; Demidchik and Shabala, 2018; Demidchik et al., 2018). The association of ROS and Ca^2+^ has been newly defined, although the relationship between them remains elusive. It was found that not only ROS has been reported to regulate Ca^2+^ channels activity (Demidchik, 2015; Choi et al., 2017), but Ca^2+^ could also induce ROS generation by activating NADPH-oxidase simultaneously (Kobayashi et al., 2007; Yamauchi et al., 2017). The relationship among ROS, Ca^2+^ and phytohormone were complex and still obscure, all of them were found to function during leaf senescence process, although more and more studies were reported in recent years, therefore, the coordination among them in the regulation of leaf senescence was more complex.

## Kinases Involved in Plant Immunity and Leaf Senescence

Among the evolutionarily conserved pathways, the mitogen-activated protein kinase (MAPK) cascade signaling pathways have been identified as important regulators of development and environmental responses in plants, especially plant immunity (Zhang et al., 2018). A typical MAPK cascade consists of at least three sequentially acting serine/threonine kinases, a MAP kinase kinase kinase (MAPKKK), a MAP kinase kinase (MAPKK) and finally, a MAP kinase (MAPK), with each phosphorylating, and hence activating the next kinase in the cascade. MAPK modules are activated in response to extracellular and/or intracellular signals and play key roles in the transduction of environmental and developmental signals through phosphorylation of downstream signaling targets, ultimately triggering major changes in gene expression and adaptive physiological responses. MAPK targets include kinases, enzymes, cytoskeletal proteins and transcription factors (Xu and Zhang, 2015; Krysan and Colcombet, 2018). There are about 80 MAPKKKs, 10 MAPKKs, and 20 MAPKs in Arabidopsis, some of which being involved in several signaling networks having an integrative function in the plants response to their environment (Chardin et al., 2017; Jagodzik et al., 2018). A variety of transcriptome analysis revealed a large number of MAPKs kinases with altered expression patterns during leaf senescence (Buchanan-Wollaston et al., 2003; Guo et al., 2004; Breeze et al., 2011; Guo and Gan, 2012). An Arabidopsis MAPKKK, MEKK1 (MAP kinase or ERK kinase kinase 1) affects leaf senescence by binding with an important senescence transcription factor WRKY53 (Miao et al., 2007), while the MEKK1-MKK1/2-MPK4 cascade negatively regulates innate immune responses (Gao et al., 2008; Kong et al., 2012); Another Arabidopsis MAPKKK kinase, EDR1 (enhanced disease resistance 1), plays a negative role in powdery mildew resistance and ethylene induced leaf senescence (Tang and Innes, 2002); the rice MAPKKK, SLES (spotted leaf sheath) is involved in disease resistance and leaf senescence by regulating the dynamic balance of ROS (Lee et al., 2018). The MKK9-MPK6 cascade in Arabidopsis positively regulates leaf senescence (Zhou et al., 2009), and also have a role in melatonin-mediated innate immunity (Lee and Back, 2016). The Arabidopsis MKK4/5-MPK1/2 cascade mediates salicylic acid induced leaf senescence (Zhang et al., 2020), while MEKK1-MKK4/5-MPK6 is activated by bacterial and fungal pathogens (Asai et al., 2002). MPK6 participates in jasmonate and salicylic acid induced plant senescence (Yue et al., 2012; Chai et al., 2014; Zhang et al., 2016), and has a role in plant defense (Pitzschke et al., 2009; Thulasi Devendrakumar et al., 2018). In addition to the mentioned kinases, there are other MAPK cascade components involved in the regulation of senescence, although it is not clear whether they have roles in the plant immune response. For instance, Arabidopsis MAPKKK18 positively regulates aging and ABA induced senescence (Matsuoka et al., 2015). Arabidopsis MAPK1/6/7 phosphorylate TTM1 to regulate its function and turnover of TTM1 during ABA triggered leaf senescence (Karia et al., 2021). Rice MAPKKK1 (SPL3, spotted leaf 3) positively regulates leaf senescence via the ABA signaling pathway (Wang S. et al., 2015). In maize, the ZmMEK1-ZmSIMK1 (*Zea mays* MAP kinase or ERK kinase-*Zea mays* salt-induced mitogen-activated protein kinase 1) cascade is involved in salicylic acid mediated leaf senescence (Li et al., 2016), while the ZmMKK10-ZmMPK3/7 cascade plays a role in ethylene-dependent cell death (Chang et al., 2017), and the ZmMPK5 kinase activity is enhanced in senescent leaves (Berberich et al., 1999). Although there are many MAPKs involved in the leaf senescence process, it is not clear how these MAPK cascades perceive and are activated by senescence signals.

**FIGURE 4:**
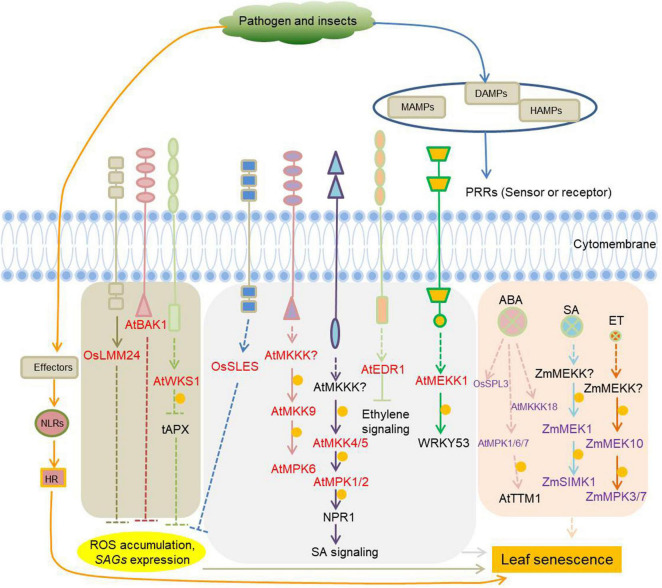
Protein kinases involved in the regulation of plant immunity-related leaf senescence. Pathogens and pests induce microbial-associated molecular patterns (MAMPs) and damage-associated molecular patterns (DAMPs) or herbivore-associated molecular patterns (HAMPs) that can be recognized by specific plant receptors (pattern recognition receptors, PRRs) to initiate cell-surface immunity. PRRs are receptor-like kinases (RLKs) or receptor-like proteins (RLPs) in plants. RLKs are composed of an extracellular ligand binding domain, a transmembrane region, and an intracellular kinase domain. RLPs have a similar structural organization to RLKs, but lack the kinase domain. Pathogens/pests can also deliver elicitors/effectors to inside of cells, and these elicitors/effectors can be sensed by intracellular immune receptors (NLRs) to initiate intracellular immunity, which will lead to hypersensitive response (HR), a form of programmed cell death (PCD). Cell-surface immunity and intracellular immunity activate downstream short-term and long-term defense responses, respectively. The kinases involved in plant immunity-mediated leaf senescence are marked in red. The kinases marked in purple are leaf senescence regulars, whether they function in response to immunity is unknown. Arrows show that the process is promoted, and the T-bars mean the process is inhibited. Yellow circles indicate phosphorylation. OsLMM24, *O. sativa* lesion mimic mutant 24; BAK1, BRI1-associated receptor kinase; WKS1, wheat kinase-start 1; tAPX, thylakoid-associated ascorbate peroxidase; OsSLES, *O. sativa* spotted leaf sheath; MKKKs or MEKKs, MAPK kinase kinases; MKKs or MEKs, MAPK kinases; NPR1, non-expresser of PR genes 1; EDR1, enhanced disease resistance 1; OsSPL3, *O. sativa* spotted leaf 3.

In addition to MAPKs, other types of kinases are jointly involved in plants defense and leaf senescence. The Rice lesion mimic mutant *lmm24*, identified as a receptor-like cytoplasmic kinase 109, is involved in the regulation of cell death and plant defense (Zhang et al., 2019). BAK1 (BRI1-associated receptor kinase), initially identified as a brassinosteroid co-receptor together with BRI1, has a much wider role as co-receptor of multiple pattern recognition receptors (PRR) involved in the regulation of cell death and plant immunity (He et al., 2007; Heese et al., 2007; Schwessinger et al., 2011; Wu et al., 2020). Increased expression of wheat stripe rust resistance protein WKS1 (wheat kinase-start 1) in transgenic wheat accelerate leaf senescence, due to the phosphorylation of the thylakoid-associated ascorbate peroxidase tAPX reducing its ability to detoxify peroxides (Gou et al., 2015). Kinases with known roles in plant defense as well as leaf senescence are listed in Figure 4 and Supplementary Table 4.

## Energy and Metabolism Associated Kinases Involved in Leaf Senescence

**FIGURE 5:**
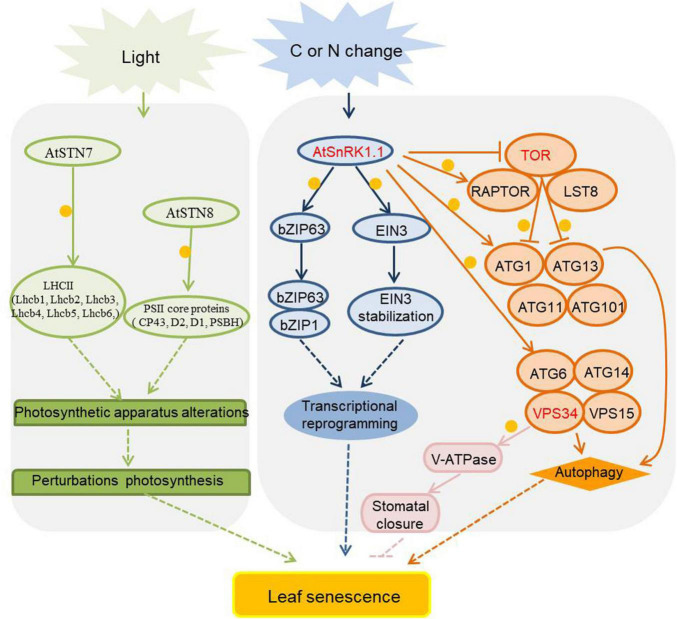
Energy and metabolism related kinases in the regulation of leaf senescence. As metabolite, carbon (C) and nitrogen (N) assimilates are transported from source organs to sink organs, and the nutrient balance of carbon and nitrogen plays an important role in signaling transduction during leaf senescence. The activities of protein kinases are affected by sugar and nitrogen signals and participate in the aging process of plants, and they are marked in red, orange circles indicate phosphorylation. Arrows show that the process is promoted, and T-bars mean the process is inhibited. The activity of SnRK1 is induced by starvation, and it phosphorylates some transcription factors to regulate *SAGs* expression or to induce the autophagy process by phosphorylating ATG1 and ATG6. SnRK1 also phosphorylates RAPTOR to inhibit the TOR’s activity. TOR negatively regulates autophagy process by phosphorylating ATG1 and ATG13 to inhibit the initial formation of autophagosomes. The PI3K protein VPS34 phosphorylates V-ATPase to activate stomatal acidification and promote stomatal closure during JA-induced leaf senescence. STN7 and STN8 maintain the balance of photosystems by phosphorylating PSII core and LHCII protein, when the phosphorylation status changed drastically, the balance would be disturbed and the leaves would senescence. STN7/8, state transitions 7/8; PSII, Photosystem II; LHCII, light-harvesting complex II; SnRK1, sucrose non-fermenting 1 related protein kinase 1; EIN3, ethylene-insensitive 3; TOR, target of rapamycin; RAPTOR, regulatory-associated protein of mTOR; LST8, lethal with SEC13 protein 8; PI3K, phosphoinositide 3-kinase; ATGs, autophagy-related proteins; VPS34, vacuolar protein sorting 34; VPS15, vacuolar protein sorting 15; V-ATPase, vacuolar H^+^-ATPase.

As sessile organisms, plants have to endure many environmental changes, which may deplete their energy stores. To survive such challenges, plants possess many energy sensors to maintain energy homeostasis. Among the energy sensors, there are a number of kinases with important roles in the regulation of plant growth, development, and stress tolerance (Doorn, 2008), including SnRK1 (sucrose non-fermenting 1 related protein kinase 1), TOR (the target of rapamycin), ATGs (autophagy-related proteins), etc. Energy and metabolism-related kinases involve in the regulation of leaf senescence are listed in Figure 5 and Supplementary Table 5.

SnRK1 is one of the evolutionarily conserved energy sensor proteins in plants. Upon activation by sugar starvation or energy depletion in cells, SnRK1 phosphorylates downstream key enzymes and induce extensive changes in gene expression patterns (Broeckx et al., 2016). There are two *SnRK1* genes in Arabidopsis, *SnRK1.1* (also known as *KIN10* or *AKIN10*) and *SnRK1.2* (also known as *KIN11* or *AKIN11*). Transgenic Arabidopsis plants overexpressing *SnRK1.1* display delayed flowering time and leaf senescence (Baena-González et al., 2007; Cho et al., 2012), however, overexpression of *SnRK1.2* leads to flower early (Williams et al., 2014). The Arabidopsis transcription factor bZIP63 plays a positive role in dark-induced senescence, and its function is repressed by SnRK1.1-mediated phosphorylation during starvation-induced senescence (Mair et al., 2014). The Arabidopsis SnRK1.1 plays a negative role in the ethylene-induced senescence process by phosphorylating the important transcription factor in ethylene signaling EIN3 (ethylene-insensitive 3) leading to its destabilization (Kim et al., 2017). The maize *SnRK1* gene family is composed of three functional members, *ZmSnRK1.1*, *ZmSnRK1.2*, and *ZmSnRK1.3*. Overexpression of all *ZmSnRK1s* in Arabidopsis results in delayed leaf senescence (Wang et al., 2019). The negative role of plant SnRK1 proteins in the regulation of leaf senescence maybe a strategy for plants to maintain cell viability and avoid sudden death under unfavorable conditions.

Target of rapamycin (TOR), an atypical Ser/Thr protein kinase that belongs to the phosphoinositide 3-kinase-related kinase family, is a central coordinator of nutrient, energy, hormone and stress signaling networks in plants (Ren et al., 2011). TOR forms kinase complexes with regulatory proteins, and these TOR interacting partners play a role in recruiting and regulating diverse TOR substrates. The TOR kinase complex comprises TOR, RAPTOR (regulatory-associated protein of TOR), and LST8 (lethal with SEC13 protein 8) in plants. There is one *TOR gene*, two *Raptor* (*RaptorA*, *RaptorB*) genes, and two *LST8* (*LST8-1, LST8-2*) genes in Arabidopsis (Xiong and Sheen, 2014). The members of the TOR complex are vital for integrating internal and external cues to regulate plant growth and development. TOR null mutants are embryo lethal; inducible RNA interference lines are small leaf size, shorter root length, early senescence, and low seed production, while *TOR*-overexpressing plants display the opposite phenotypes (Deprost et al., 2007; Ren et al., 2011). *lst8-1* mutants show modest dwarf growth and accelerated senescence (Moreau et al., 2012).

Plant autophagy is a highly conserved catabolic process in which cells encapsulate and deliver cytoplasmic components into the vacuole for degradation and recycling of essential nutrients (Li and Vierstra, 2012; Liu and Bassham, 2012). Autophagy is primarily induced by natural senescence and a variety of unfavorable environmental factors, which will lead to nutrient limitation and accelerated nutrient recycle, e.g., nutrient deprivation, high salt, drought, hypoxia, oxidative stress, pathogen infection (Marshall and Vierstra, 2018a). There are a large number of autophagy-related proteins (ATGs) in plants with essential roles in regulation of autophagy (Soto-Burgos et al., 2018; Yoshimoto and Ohsumi, 2018). Plant ATG complexes are grouped into four functional categories: (1) proteins that initiate autophagy, including the ATG1 kinase core complex, containing four subunits: ATG1/ATG13/ATG17-ATG29-ATG31/ATG11; (2) proteins that mediate emergence of phagophores, including the ATG9 kinase complex, containing three subunits: ATG9/ATG2/ATG18; (3) factors that remodel autophagic membranes, including the class III phosphatidylinositol-3-kinase (PI3K) complex, containing the VPS34 (Vacuolar protein sorting 34), VPS15, ATG6 and ATG14 four subunits; (4) two ubiquitin-like conjugation complexes, ATG5-ATG12 and ATG8-PE (phosphatidylethanolamine), which decorate phagophores and autophagosomes (Marshall and Vierstra, 2018b; Marshall et al., 2019). Arabidopsis ATG mutants display premature leaf senescence and shortened life cycle even under normal growth conditions, hypersensitivity to nutrient deficiency, decreased tolerance to biotic and abiotic stresses, activated innate immunity, and an altered cellular metabolism (Doelling et al., 2002; Xiong et al., 2007; Liu et al., 2009; Hayward and Dinesh-Kumar, 2011; Guiboileau et al., 2012; Li et al., 2014; Avin-Wittenberg et al., 2015; Qi et al., 2020). Interestingly, both the TOR kinase and SnRK1 are involved in autophagy by phosphorylation of ATGs. Under nutrient-rich conditions, TOR phosphorylates the ATG13 and ATG1 subunits to prevent autophagy. Meanwhile, in nutrient starvations conditions SnRK1.1 phosphorylates ATG1 and ATG6 to induce autophagy (Chen et al., 2017; Pu et al., 2017; Soto-Burgos and Bassham, 2017; Huang et al., 2019). Moreover, PI3K interacts with V-ATPase (vacuolar H^+^-ATPase) to activate stomatal acidification, which leads to stomatal closure and delayed leaf senescence, and also alleviates leaf senescence under jasmonate treatment (Liu et al., 2016a,b).

STN7 and STN8 (state transitions 7/8) are important chloroplast kinases that can phosphorylate different photosynthesis-associated thylakoid proteins to adapt to environmental changes (Bellafiore et al., 2005; Bonardi et al., 2005). The primary function of STN7 is the phosphorylation of LHCII (light-harvesting complex II) triggering its migration to PSI (photosystem I) to initiate a state transition. STN8 phosphorylates PSII (photosystem II) core proteins to modulate thylakoid ultrastructure and facilitates the repair of damaged PSII. STN7 and STN8 help to maintain optimal activity of the photosynthetic apparatus and have a crucial role in short-term acclimation and long-term responses (Vainonen et al., 2005; Puthiyaveetil et al., 2012; Poudyal et al., 2020). Interestingly, both loss-of-function and overexpression of *STN7* and *STN8* result in early onset of senescence, suggesting that any perturbations of these two genes-regulated acclimation processes will induce early senescence in plants (Wang et al., 2015).

## Conclusion and Perspectives

It is an important approach to reveal molecular mechanism of leaf senescence by investigating genetic mutants with altered leaf senescence process. Many kinase-associated mutants and/or transgenic plants were detected earlier/delayed leaf senescence phenotype and these materials played roles to find new components and their regulatory networks involved in the leaf senescence process (Li et al., 2020). A large number of *SAGs* have been found by differential expression techniques in different plants (Guo et al., 2004; Buchanan-Wollaston et al., 2005; Breeze et al., 2011; Guo and Gan, 2012), and some SAGs were protein kinases, which play roles in signal transduction during leaf senescence. The researchers have found lots of SAPs (senescence associated proteins) through proteomics approaches and combined the information of metabolites change by metabolomics during leaf senescence, however, no protein kinase was detected as SAPs because of their low abundance in nature (Hebeler et al., 2008; Watanabe et al., 2013; Balazadeh et al., 2014; Moschen et al., 2016; Wei et al., 2016). Phosphoproteomic data identified many phosphorylation motifs, and it showed us potential kinase-substrate or kinase phosphorylation site during leaf senescence, moreover, the information of co-expression kinases and external co-localization or co-interaction were also supplied by phosphoproteomic data (Mergner et al., 2020), further work to find new protein kinase in the regulation of leaf senescence or study the function of protein kinase in leaf senescence by taking advantage of this data. Despite many kinases as the senescence regulators have been found involved in leaf aging, the substrates of most leaf aging-related kinases are still unknown, which is vital to discover the entire signaling cascades or pathway during leaf senescence. The proteome and metabolite profiling analyses are effective approaches to expand and verify transcriptomics-induced molecular responses. Integration of multi-omics data including genomic, transcriptomic, proteomic, and metabolomic of leaf senescence wound provide a possible pathway to find the potential kinase-substrate combination during leaf senescence and reveal their molecular function. Therefore, considering the importance and complexity of signaling pathway in leaf senescence, and the vital roles of protein kinases in signal transduction, the in-depth study on leaf senescence using integrated omics approaches would help to unravel the key issues in leaf senescence, such as how and when plant initiate, execute and finish leaf senescence process, what is the initiate signal of leaf senescence, what are the differences between natural leaf senescence and stress-induced leaf senescence. Finally, studies on screening and functional analysis of senescence associated kinases are directly linked with growth and breeding, it is the cornerstone for improving crop production.

Phosphorylation by protein kinases has a strong effect on the conformation, activity, stability, and localization of target proteins. Leaf senescence is an integral part of plant development, and it is affected by internal and external factors. The different kinases involved in the regulation of leaf senescence play vital roles in the perception of senescence-associated information and transmission of the signal to downstream factors. Although a large number of kinases have been implicated in the regulation of plant leaf senescence, further work is needed to build the connections between the different components of the senescence process, and novel signaling components and pathways will continue to be discovered. Elucidation of the senescence mechanisms associated with environmental fitness and reproduction could be used to enhance stress tolerance and improve crop yield.

## Author Contributions

FY, KL, and C-PS wrote the manuscript. YM, WL, YL, and JB commented on the first draft and critically reviewed the final manuscript. All authors contributed to the article and approved the submitted version.

## Conflict of Interest

The authors declare that the research was conducted in the absence of any commercial or financial relationships that could be construed as a potential conflict of interest.

## Publisher’s Note

All claims expressed in this article are solely those of the authors and do not necessarily represent those of their affiliated organizations, or those of the publisher, the editors and the reviewers. Any product that may be evaluated in this article, or claim that may be made by its manufacturer, is not guaranteed or endorsed by the publisher.
