# Supplementary material for: Function of Protein Kinases in Leaf Senescence of Plants
Source: Front Plant Sci. 2022 Apr 25;13:864215. doi: 10.3389/fpls.2022.864215 (PMC9083415; doi:10.3389/fpls.2022.864215)
Supplement: Supplementary file 1 [file Table_1.docx]

Supplementary table 1. Kinases function in plant hormone related leaf senescence.

| **Kinase Name** | **Species** | **Performance during leaf senescence** | **Function** | **Role** | **Reference** |
| --- | --- | --- | --- | --- | --- |
| EDR1 | *A. thaliana* | *edr1* mutants show enhanced leaf senescence under ethylene treatment | A MAPKKK, plays a negative role in the ethylene signaling pathway | Negative | Tang and Innes, 2002; Tang et al., 2005 |
| SnRK2s | *A. thaliana* | Mutants are insensitive to ABA-induced leaf senescence | Phosphorylate ABFs and RAV1 to activate the expression of *SAGs* in ABA-induced leaf senescence | Positive | Zhao et al., 2014; Gao et al., 2016 |
| MPK6 | *A. thaliana* | *mpk6* shows delayed aging symptoms under jasmonic acid (JA)- and salicylic acid (SA)- treatment | Involves in both JA- and SA- mediated leaf senescence | Positive | Yue et al., 2012; Chai et al., 2014; Zhang et al., 2016 |
| MKK4/5 | *A. thaliana* | Mutants show reduced aging symptoms under SA treatment | Regulates SA-induced leaf senescence through phosphorylation of MPK1/2-NPR1 | Positive | Zhang et al., 2020 |
| MPK1/2 | *A. thaliana* | Mutants display reduced aging symptoms under SA treatment | Regulates SA-induced leaf senescence through phosphorylation of NPR1 | Positive | Zhang et al., 2020 |
| BRI1 | *A. thaliana* | Mutants show dark-green leaves and delayed senescence | Encodes a plasma membrane localized leucine-rich repeat receptor kinase, as BR receptor | Positive | He et al., 2007 |
| AHKs | *A. thaliana* | Mutants show shorter leaf longevity and loss of the ability to retain chlorophyll under cytokinin (CK) treatment in dark-induced leaf senescence | Arabidopsis histidine kinases, CK receptors | Negative | Riefler et al., 2006 |
| SARK | *A. thaliana* | *AtSARK*- overexpressing seedlings display precocious leaf senescence | Regulates leaf senescence through synergistic actions of auxin and ethylene; SAURs (SMALL AUXIN-UP RNA proteins) accelerate leaf senescence process via the activation of SARK-mediated signaling by suppressing of SSPP | Positive | Xu et al., 2011; Wen et al., 2020 |
