# Supplementary material for: Function of Protein Kinases in Leaf Senescence of Plants
Source: Front Plant Sci. 2022 Apr 25;13:864215. doi: 10.3389/fpls.2022.864215 (PMC9083415; doi:10.3389/fpls.2022.864215)
Supplement: Supplementary file 2 [file Table_2.docx]

Supplementary table 2. Kinases involved in ROS signal and leaf senescence.

| **Kinase Name** | **Species** | **Performance during leaf senescence** | **Function** | **Role** | **Reference** |
| --- | --- | --- | --- | --- | --- |
| AtABC1K1/3 | *A. thaliana* | *abc1k1* and *abc1k3* mutants display rapid chlorosis under high light stress, and double mutants show slow and irreversible senescence-like phenotype under moderate light | ABC1K1/3 regulate chlorophyll degradation by jasmonate biosynthesis and pheophytinase activity, and oxidative stress response | Negative | Lundquist et al., 2013 |
| OsABC1-2 | *O. sativa* | *osabc1-2* show small plant size and pale-green leaves, and the *OsABC1-2* overexpressing lines show enhanced tolerance to prolonged dark-induced leaf senescence | ABC1 atypical kinase is located in either chloroplasts or mitochondria, response to stresses | Negative | Gao et al., 2012 |
| AtABC1K7/8 | *A. thaliana* | *abc1k7*, *abc1k8* and *abc1k7 abk1k8* mutants exhibit faster senescence rate than WT plant under ABA treatment | ABC1K7 and ABC1K8 are involved in ROS homeostasis and upregulated by ABA | Negative | Manara et al., 2016 |
| OXI1 | *A. thaliana* | *OXI1* over-expressing lines display hypersensitivity to high light and early senescence even in normal light conditions | A component of ROS signals and is activated by oxidative stress and wounding | Positive | Shumbe et al., 2016; Beaugelin et al., 2019 |
| MEKK1 | *A. thaliana* | None available | [MEKK1 [mediates ROS homeostasis](https://pubmed.ncbi.nlm.nih.gov/17043356/); MEKK1 directly interacts and activates the senescence-related WRKY53 and it also binds to the promoter of *WRKY53*](https://pubmed.ncbi.nlm.nih.gov/17587183/) | Positive | Nakagami et al., 2006; Miao et al., 2007 |
| MPK6 | *A. thaliana* | The null *MPK6* mutants show delay leaf senescence | MPK6 is induced by oxidative stress | Positive | Zhou et al., 2009 |
