# Supplementary material for: Function of Protein Kinases in Leaf Senescence of Plants
Source: Front Plant Sci. 2022 Apr 25;13:864215. doi: 10.3389/fpls.2022.864215 (PMC9083415; doi:10.3389/fpls.2022.864215)
Supplement: Supplementary file 3 [file Table_3.docx]

Supplementary table 3. Kinases involved in Ca^2+^ signal and leaf senescence.

| **Kinase Name** | **Species** | **Performance during leaf senescence** | **Function** | **Role** | **Reference** |
| --- | --- | --- | --- | --- | --- |
| BnaCPK6L | *B. napus* | None available | Interacts and phosphorylates BnaRBOHD to enhance BnaRBOHD activity and generate more ROS in cell which would accelerate cell death and leaf senescence | Positive | Pan et al., 2019 |
| BnaCPK2 | 1. *napus* | None available | Interacts and phosphorylates BnaRBOHD to enhance BnaRBOHD activity and generate more ROS in cells | Positive | Wang et al., 2018 |
| BnaCPK5/6 | *B. napus* | None available | Interact and phosphorylate BnaWSR1 to enhance its transcriptional activity, regulate cell death and leaf senescence | Positive | Cui et al., 2020 |
| OsESL4 | *O. sativa* | *esl4* mutants show premature leaf senescence under low-nitrogen conditions | Regulats leaf senescence by affecting ROS levels and photosynthetic rate | Negative | Xing et al., 2018 |
| OsCPK12 | *O. sativa* | Impaired function of the OsCPK12 leads to early senescence in rice | The activities of CAT, POD and soluble protein content in mutants were lower than WT, while the content of ROS and MDA were higher than WT | Negative | Wang et al., 2019 |
| ZmCPK11 | *Z. mays* | Overexpression of *ZmCPK11* in Arabidopsis delayed the salt-induced leaf senescence | Enhances salt tolerance by preventing salt-induced chlorophyll degradation and damage to photosystem II | Negative | Borkiewicz et al., 2020 |
| AtCRK3 | *A. thaliana* | None available | AtCRK3 could be induced by natural or artificially induced leaf senescence; phosphorylates the cytosolic glutamine synthetase AtGLN1;1/AtGSR1, which is important for nitrogen remobilization and reutilization during leaf senescence | Unknown | Li et al., 2006 |
| AtCIPK14 | *A. thaliana* | The mutants display delayed leaf senescence and the overexpression lines show the opposite phenotypes to mutants | Phosphorylates WHY1 and increased the accumulation of WHY1 in nucleus, promotes its binding to the promoter of *WRKY53* and thus inhibits the expression of several *SAGs* | Negative | Ren et al., 2017 |
