# Supplementary material for: Function of Protein Kinases in Leaf Senescence of Plants
Source: Front Plant Sci. 2022 Apr 25;13:864215. doi: 10.3389/fpls.2022.864215 (PMC9083415; doi:10.3389/fpls.2022.864215)
Supplement: Supplementary file 4 [file Table_4.docx]

Supplementary table 4. Protein kinases involved in the regulation of plant immunity-related leaf senescence.

| **Kinase Name** | **Species** | **Performance during leaf senescence** | **Function** | **Role** | **Reference** |
| --- | --- | --- | --- | --- | --- |
| OsLMM24 | *O. sativa* | *lmm24* show enhanced ROS accumulation and cell death, decreased expression of photosynthesis-related genes and increased expression of the senescence-induced *STAYGREEN* (*SGR*) gene and other *SAGs* | As a receptor-like cytoplasmic kinase, OsLMM24 is involved in the regulation of cell death and plant defense. *lmm24* exhibited enhanced resistance to rice blast fungus *Magnaporthe oryzae* and up-regulated of defense response genes | Negative | Zhang et al., 2019 |
| AtBAK1 | *A. thaliana* | RNA interference plants shows severe growth retardation and early senescence | As a brassinosteroid co-receptor and together with BRI1, regulates cell death and plant immunity | Negative | He et al., 2007; Heese et al., 2007;  Schwessinger et al., 2011; Wu et al., 2020 |
| *Ta*WKS1 | *T. aestivum* | Increased expression of *WKS1* accelerates leaf senescence | phosphorylates the thylakoid-associated ascorbate peroxidase tAPX and reduces its ability to detoxify peroxides | Positive | Gou et al., 2015 |
| OsSLES | *O. sativa* | *sles* plants show early yellowing compared with WT | SLES is involved in disease resistance and leaf senescence by regulating the dynamic balance of ROS | Negative | Lee et al.,  2018 |
| AtMKK9 | *A. thaliana* | *mkk9* plants show delayed yellowing compared with WT, and the overexpression lines have the opposite phenotype to mutants | The MKK9-MPK6 cascade in Arabidopsis positively regulates leaf senescence | Positive | Zhou et al., 2009 |
| AtMPK6 | *A. thaliana* | *mkp6* plants show delayed yellowing compared with WT in natural development, jasmonate and SA treatment, and the overexpression lines show opposite phenotype to mutants | Participates in natural, jasmonate and SA induced plant senescence | Positive | Yue et al., 2012; Chai et al., 2014; Zhang et al., 2015; Liu et al., 2016 |
| AtMKK4/5 | *A. thaliana* | *mkk4/5* plants showed delayed yellowing compared with WT under SA treatment, and the overexpression lines display opposite phenotype to mutants | Regulate SA-induced leaf senescence through phosphorylation of NPR1 | Positive | Zhang et al., 2020 |
| AtMPK1/2 | *A. thaliana* | *mpk1/2* plants show delayed yellowing compared with WT under SA treatment, and the overexpression lines display opposite phenotype to mutants | Regulates SA-induced leaf senescence through phosphorylation of NPR1 | Positive | Zhang et al., 2020 |
| AtEDR1 | *A. thaliana* | *edr1* mutants show enhanced leaf senescence under ethylene treatment | A MAPKKK, plays a negative role in the regulation of ethylene signaling pathway | Negative | Tang and Innes, 2002; Tang et al., 2005 |
| AtMEKK1 | *A. thaliana* | None available | Affects leaf senescence by binding to the promoter of WRKY53 | Positive | Miao et al., 2007 |
| OsSPL3 | *O. sativa* | *spl3* mutant shows a stay-green phenotype during ABA-induced leaf senescence | Positively regulates leaf senescence via the ABA signaling pathway; function in immunity is unknown | Positive | Wang et al., 2015 |
| AtMPK1/6/7 | *A. thaliana* | MPK1/7 is None available, *mkp6* plants show delayed yellowing compared with WT in natural development, jasmonate and SA treatment, and the overexpression lines show opposite phenotype to mutants | Phosphorylate TTM1 (triphosphate tunnel metalloenzyme) to regulate its function and turnover of TTM1 during ABA triggered leaf senescence; MPK1/7 function of immunity is unknown | Positive | Karia et al., 2021 |
| AtMKKK18 | *A. thaliana* | *mkkk18* mutant shows a stay-green phenotype during natural and ABA-induced leaf senescence | Positively regulates aging and ABA induced senescence; function in immunity is unknown | Positive | Matsuoka et al., 2015 |
| ZmMEK1 | *Z. mays* | Inactive ZmMEK1 causes early leaf senescence in transgenic Arabidopsis under normal condition and SA treatment | ZmMEK1-ZmSIMK1 cascade is involved in salicylic acid mediated leaf senescence; function in immunity is unknown | Positive | Li et al., 2016 |
| ZmSIMK1 | *Z. mays* | None available | ZmMEK1-ZmSIMK1 cascade is involved in SA mediated leaf senescence; function in immunity is unknown | Positive | Li et al., 2016 |
| ZmMEK10 | *Z. mays* | Expression of the active ZmMKK10 induces cell death in transgenic Arabidopsis | ZmMKK10-ZmMPK3/7 cascade plays a role in ethylene-dependent cell death; function in immunity is unknown | Positive | Chang et al., 2017 |
| ZmMPK3/7 | *Z. mays* | None available | ZmMKK10-ZmMPK3/7 cascade plays a role in ethylene-dependent cell death; function in immunity is unknown | Positive | Chang et al., 2017 |
