# Supplementary material for: Function of Protein Kinases in Leaf Senescence of Plants
Source: Front Plant Sci. 2022 Apr 25;13:864215. doi: 10.3389/fpls.2022.864215 (PMC9083415; doi:10.3389/fpls.2022.864215)
Supplement: Supplementary file 5 [file Table_5.docx]

Supplementary table 5. Kinases involved in energy and nutrient recycling and leaf senescence.

| **Kinase Name** | **Species** | **Performance during leaf senescence** | **Function** | **Role** | **Reference** |
| --- | --- | --- | --- | --- | --- |
| AtSTN7 | *A. thaliana* | Both the mutants and overexpression plants show early senescence phenotype | Phosphorylates of LHCII (light-harvesting complex II) and triggers its migration to PSI (photosystem I) to initiate a state transition | unknown | Bonardi et al., 2005; Wang et al., 2015; Poudyal et al., 2020 |
| AtSTN8 | *A. thaliana* | Both the mutants and overexpressors show early senescence phenotypes | STN8 phosphorylates PSII (photosystem II) core proteins to modulate thylakoid ultrastructure and facilitates the repair of damaged PSII | unknown | Bellafiore et al., 2005; Wang et al., 2015 |
| AtSnRK1.1 | *A. thaliana* | Overexpression plants display delayed flowering time and leaf senescence in age-dependent senescence; overexpression plants display delayed leaves yellowing under dark-induced and ethylene-induced leaf senescence | SnRK1.1 phosphorylates and inhibites bZIP63 during starvation-induced senescence;  SnRK1.1 plays a negative role in ethylene-induced senescence by phosphorylating EIN3 (ethylene-insensitive 3) and leads to its destabilization | Negative | Cho et al., 2012; Mair et al., 2015; Kim et al., 2017 |
| ZmSnRK1s | *Z. mays* | Overexpression of all ZmSnRK1s in Arabidopsis results in delayed leaf senescence | Senescence-associated genes, such as SAG13 and WRKY53, which are positive regulators of leaf senescence, were down-regulated in the transgenic lines ZmSnRK1s overexpression Arabidopsis inhibits the expression of *SAG13* and *WRKY53*, which are positive regulators of leaf senescence | Negative | Wang et al., 2019 |
| TOR | *A. thaliana* | *tor* null mutants are embryo lethal; inducible RNA interference lines show small leaf size, shorter root length, early senescence, and low seed production, while *TOR*-overexpression plants also display early aging phenotypes | *TOR* silenced leaves show higher concentrations of soluble sugars, higher activity of glutamine synthetase and glutamate dehydrogenase , and TOR activity is needed for restrain senescence and nutrient recycling | Unknown | Deprost et al., 2007; Ren et al., 2011 |
| VPS34 | *A. thaliana* | None available | The inhibition of VPS34 suppressed the vacuolar acidification and enhanced the stomatal opening, thereby accelerating MeJA-induced leaf senescence. | Negative | Marshall et al., 2019 |
